# Diverticular disease management in primary care: How do estimates from community-dispensed antibiotics inform provision of care?

**DOI:** 10.1371/journal.pone.0219818

**Published:** 2019-07-17

**Authors:** Joanna B. Broad, Zhenqiang Wu, Jerome Ng, Bruce Arroll, Martin J. Connolly, Rebekah Jaung, Frances Oliver, Ian P. Bissett

**Affiliations:** 1 Department of Geriatric Medicine, University of Auckland, Auckland, New Zealand; 2 Institute for Innovation and Improvement, Waitematā District Health Board, Auckland, New Zealand; 3 Department of General Practice and Primary Healthcare, School of Population Health, University of Auckland, Auckland, New Zealand; 4 Waitematā District Health Board, Auckland, New Zealand; 5 Department of Surgery, University of Auckland, Auckland, New Zealand; University of Campania, ITALY

## Abstract

**Background:**

The literature regarding diverticular disease of the intestines (DDI) almost entirely concerns hospital-based care; DDI managed in primary care settings is rarely addressed.

**Aim:**

To estimate how often DDI is managed in primary care, using antibiotics dispensing data.

**Design and setting:**

Hospitalisation records of New Zealand residents aged 30+ years during 2007–2016 were individually linked to databases of community-dispensed oral antibiotics.

**Method:**

Patients with an index hospital admission 2007–2016 including a DDI diagnosis (ICD-10-AM = K57) were grouped by acute/non-acute hospitalisation. We compared use of guideline-recommended oral antibiotics for the period 2007–2016 for these people with ten individually-matched non-DDI residents, taking the case’s index date. Multivariable negative binomial models were used to estimate rates of antibiotic use.

**Results:**

From almost 3.5 million eligible residents, data were extracted for 51,059 index cases (20,880 acute, 30,179 non-acute) and 510,581 matched controls; mean follow-up = 8.9 years. Dispensing rates rose gradually over time among controls, from 47 per 100 person-years (/100py) prior to the index date, to 60/100py after 3 months. In comparison, dispensing was significantly higher for those with DDI: for those with acute DDI, rates were 84/100py prior to the index date, 325/100py near the index date, and 141/100py after 3 months, while for those with non-acute DDI 75/100py, 108/100py and 99/100py respectively. Following an acute DDI admission, community-dispensed antibiotics were dispensed at more than twice the rate of their non-DDI counterparts for years, and were elevated even before the index DDI hospitalisation.

**Conclusion:**

DDI patients experience high use of antibiotics. Evidence is needed that covers primary-care and informs self-management of recurrent, chronic or persistent DDI.

## Introduction

Diverticular disease of the intestine (DDI) is a common long term condition with significant impacts on morbidity[1–4] and healthcare expenditure.[5, 6] Thorough understanding of the epidemiology and current management patterns is important for optimal management,[7, 8] although research about DDI–its epidemiology and treatment–almost entirely uses hospitalisation data.[9–12] The frequency that DDI is managed in primary care is largely unknown. In the Netherlands, most patients presenting to primary care with symptoms suggestive of DDI are managed in the community; just one in eight patients is hospitalised.[13] In Italy, a general practice research database shows DDI incidence across all ages as about 2.6 per 1000, of whom 27% had been hospitalised since, but just 3.4% were admitted for DDI.[14] Variations in current practice and lack of consensus with clinical guidelines indicate the potential for new evidence to enable improvements in care and advice.[14–17] Not the least of these variations concerns use of antibiotics–when is their use necessary, which medications to choose, and can their use be avoided in order to reduce antibiotic resistance?[18] Until quite recently, oral antibiotics (e.g. penicillin) have been the mainstay of non-surgical DDI management.[19] Therefore, one potential approach to estimate the impact of DDI in the community is to examine and compare the patterns of antibiotic use between people with a history of DDI against matched counterparts without the disease. Antibiotic use that exceeds that of population controls has potential to indicate the extent DDI is managed in primary care. Using this approach, this study aims to use any excess in antibiotic dispensing to estimate how often DDI is managed in primary care.

## Methods

The University of Auckland Human Participants Ethics Committee approved the study (Ref. 016560).

### Study design and data sources

In New Zealand (NZ), Ministry of Health (MoH) collects details of publicly-funded hospitalisations, subsided prescribed medicines and deaths, visits and subsidy claims in primary care but does not collect clinical information. In this population-based study we assembled relevant antibiotic dispensing records for a whole population, comparing dispensing for people with a prior hospital-based DDI diagnosis with that of their counterparts without a DDI history. Unlike some other countries, in New Zealand antibiotics are available only when prescribed by a registered medical practitioner and dispensed by a qualified and registered pharmacist.

Three linked national health databases are employed: the National Health Index (NHI), the National Minimum Dataset (NMDS), and the Pharmaceutical database (PHARMS). The NHI contains demographic and identifying information for all who use health and disability support services in the health system, and covers at least 95% of the NZ population.[20] The NMDS collects public and private hospital discharge data for inpatients and day patients (excluding those presenting to emergency departments or if discharged within 3 hours).[21] Since July 1999 (throughout this study), hospital diagnoses have been coding using the ICD-10-AM system.[22] Each hospitalisation is also classified by clinical coders as acute or non-acute (booked or waitlisted) using an “admission type” code. The PHARMS database collects community-based dispensing data for prescribed and subsidised medications.[23] All three databases are indexed with an encrypted patient identifier. Data from all sources were extracted for each eligible resident and merged individually.

### Selection and classification of participants

**Fig 1:**
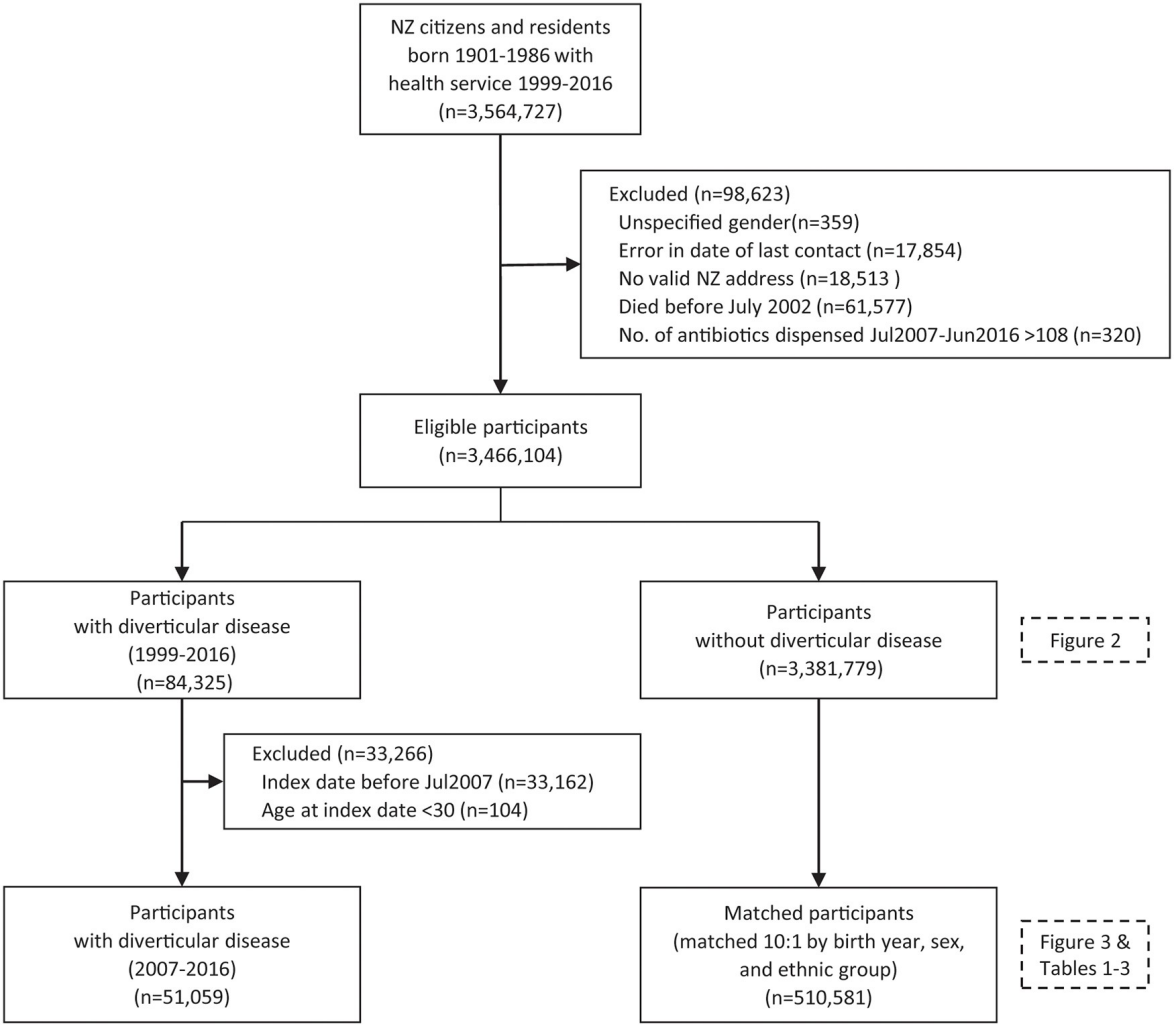
Flow chart of included people with diverticular disease of the intestine and matched people without diverticular disease.

Study participants were selected from the NHI database if meeting the following criteria: were citizens or residents of NZ; born between 1900 and 1986; date of last contact with the health service–hospitalisation, an outpatient visit, a delivery (as mother), a death, a medication dispensed from a community pharmacy, a primary care consultation or enrolment, a contact with a mental health service, a vaccination, a laboratory test, or a medical subsidy–was after July 1999 and at that time they gave a NZ address; there was no record of DDI prior to 1 July 2007; and there was no record of death before June 2002. Almost all residents aged 30+ years are therefore included, except those with missing demographic data for gender, year of birth or region of current residence, or with clear errors or outliers, e.g. exceeding 108 antibiotics dispensed during the 9 year period (more than once a month), or with prior DDI (Fig 1).

From the NMDS database, hospital admissions during July 2002-June 2016 with DDI identified using any of the ICD-10-AM codes K57.0–K57.9 (whether incident/recurrent, uncomplicated/not, primary/other diagnosis or colonic/otherwise) were regarded as having DDI[24]. The first DDI admission after 30 years of age was regarded as the “index” admission, classed as acute/non-acute using a routine clinical code, and the date of that admission taken as the index date.

Non-DDI participants were randomly matched 10:1 with DDI participants of the same birth year, gender and self-identified ethnic group, taking the admission date of their matched case as their index date.

### Use of prescribed antibiotics

From the PHARMS database were extracted records of relevant government-subsidised oral antibiotics dispensed by community pharmacies 1 July 2007–30 June 2016 (Table 1). Using published articles and in consultation with NZ medical practitioners, we pre-specified a list of DDI-relevant antibiotics available from community pharmacies under NZ’s pharmaceutical schedule.[25–28] The dispensing database was searched for all participants to extract the chemical name, formulation name and date dispensed. Since it is extremely rare in NZ for government-funded medicines to be paid for entirely privately, dispensing data will be virtually complete.

**Table 1:** Number of days on which chosen antibiotics were dispensed (for any purpose) to participants with a history of diverticular disease of the intestine (n = 51,059) and matched participants (n = 510 581), over the 9-year period from July 2007 to June 2016.

Chemical name	Chemical code	Anatomical Therapeutic Chemical (ATC) classification codes	Courses dispensed	
			N	%
**Present in dispensing database**:				
Amoxicillin with clavulanic acid	1070	J01CR02	928 592	36.1
Amoxicillin without clavulanic acid	1072	J01CA04, QG51AX03	821 931	32.0
Cefaclor monohydrate	1228	J01DC04	277 689	10.8
Ciprofloxacin	2819	J01MA02, S01AE03, S02AA15, S03AA07	208 227	8.1
Co-trimoxazole	1361	J01EE01	150 361	5.9
Metronidazole	1820	A01AB17, D06BX01, G01AF01, J01XD01, P01AB01, QP51AA01	108 324	4.2
Cefalexin	1234	J01DB01, QJ51DB01	37 418	1.5
Clindamycin	1303	J01FF01, D10AF01, G01AA10	24 120	0.9
Ornidazole	1906	G01AF06, J01XD03, P01AB03, QP51AA03	14 409	0.6
Moxifloxacin	3925	J01MA14, S01AE07	218	<0.1
Total N of courses of chosen antibiotics dispensed			2 571 289	100.0
**Searched for but not found during study period**:				
Tinidazole	2269	J01XD02, P01AB02, QP51AA02		
Pivampicillin	1998	J01CA02		
Pivampicillin hydrochloride	1999	J01CA02		
Levofloxacin	3789	J01MA12, S01AE05		

Note: Other antibiotics (including rifaximin) that are used for DDI in some countries are not listed as they are not funded for diverticular disease and are rarely if ever used in NZ.

### Outcomes

Rates of dispensing (for any reason) for specified periods were calculated by dividing the number of days on which antibiotics were dispensed by the total number of days spent alive during the follow-up period. Rates among those with an index date between 2007–2016 were derived.

### Analyses

Firstly, for one exemplar 12-month period (July 2012-June 2013) the mean number of antibiotics dispensed was plotted to show annual dispensing rates by age and gender for each of three groups: non-DDI, acute DDI and on-acute DDI.

Secondly, to avoid assuming any statistical distribution across time, 21 separate negative binomial regression models were used to estimate dispensing rates, each for six-months: one centred on the index date, with 10 models prior to, and 10 models after, the index date. A plot shows the pattern of dispensing over time, centred on the index date.

Thirdly, because of the pattern seen in the above results, time was summarised into three periods and dispensing rates modelled within each: *Model A* for the period 2007 to 3 months prior to index date; *Model B* for the period +/-3 months about the index date; and *Model C* for the period 3 months after index date until 2016. Following the advice of Pearce[29] “standard” (unconditional) negative binomial regression models were employed, each to estimate three measures of antibiotic use: *dispensing rates* (DR, defined as the mean number of days on which chosen antibiotics were dispensed per 100 person-year), *dispensing rate difference* (DRD, the absolute difference between the rates of the two groups) and *dispensing rate ratio* (DRR, the relative difference between the two groups). Models adjusted for DDI history, year of birth, index year, gender, ethnic group and region.

Fourthly, sensitivity analyses were conducted similar to Models A-C to provide for the matched design by using conditional multivariable negative binomial regressions.

Statistical analyses were performed using SAS v9.4 (SAS Institute Inc., Cary, NC) except the sensitivity analyses that were conducted in Stata v13.0 (StataCorp, College Station, TX). Because of large numbers and multiple testing, two-sided p-values <0.001 were considered statistically significant.

## Results

### Participation

Of the initial 3,564,727 person records initially extracted, 98,623 were excluded, leaving 3,466,104 eligible participants (Fig 1). During the wider 17-year study period, 84,325 (2.4%) had a hospital diagnosis of DDI; by ICD code, over 97% were colonic.

During the shorter, 9-year period July 2007 to June 2016 (mean duration = 8.6 years), 51,059 (mean age 67.3 years, 23,853 men) received a hospital diagnosis of DDI. DDI was the primary diagnosis code recorded for 20,525 (40.2%); 20,880 (40.9%) were acute index admissions of whom 19,337 (93.6%) were admitted at least overnight. In the remaining 30,179 (59.1%) index admissions, 26,578 (98.3%) received a non-acute colonoscopy. For comparison purposes, 510,581 non-DDI participants were individually-matched to serve as controls.

After the index DDI hospitalisation, 9697 (19.0%) had at least one DDI re-hospitalisation during follow-up (mean 3.6 years).

### Antibiotics dispensed

**Fig 2:**
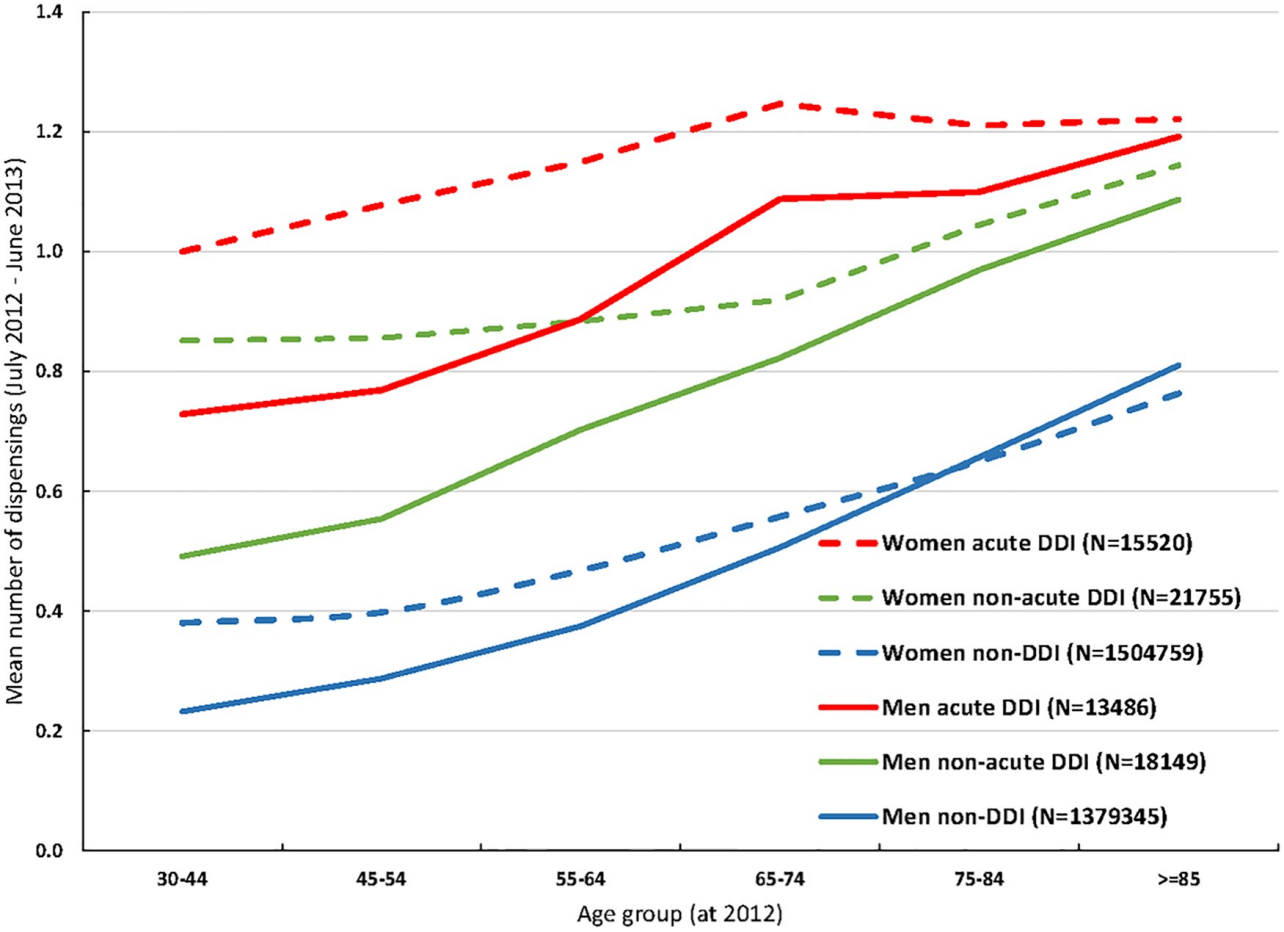
Mean number of days on which antibiotics were dispensed in a single year (July 2012 to June 2013), for those with known prior acute or non-acute hospitalisation for diverticular disease of the intestine (DDI), and those without (non-DDI). Notes: Age calculated as at 2012; DDI = hospitalisation with diverticular disease of intestines, acute or non-acute; non-DDI = controls with no hospital record of diverticular disease of intestines.

Pre-specified oral antibiotics were dispensed by community pharmacies on 2,597,464 occasions, for 47,603 DDI and 383,882 non-DDI participants, over the 9-year period (Table 1). The most commonly dispensed were amoxicillin with/without clavulanic acid, cefaclor monohydrate and ciprofloxacin, together accounting for 87% (Table 1). Over a 12 month period, numbers of community-dispensed antibiotics per person were higher for women than for men, increased with age, and highest for participants with acute DDI (Fig 2).

**Fig 3:**
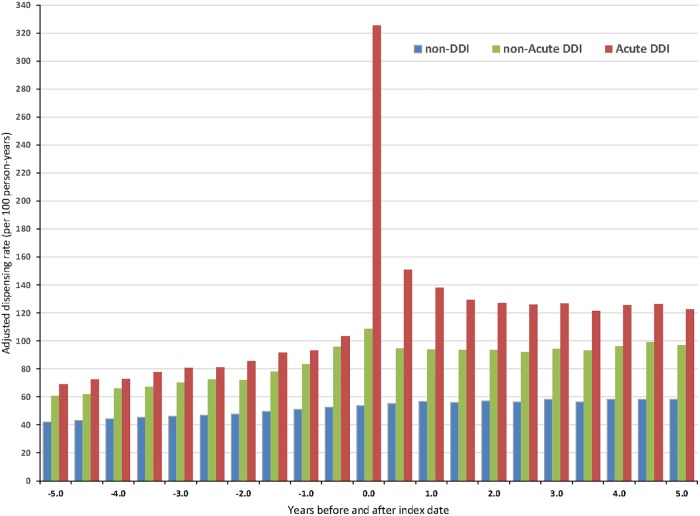
Dispensing rate of selected antibiotics before and after index date for diverticular disease of the intestine participants (n = 51,059: Acute DDI 20,880, non-acute DDI 30,179) and matched non-DDI participants (n = 510 581) during the period July 2007-June 2016.

Fig 3 illustrates dispensing rates for 6-month periods centred on the index date. During the years prior to the index date, dispensing rates for acute DDI participants rose from a low of about 70 per 100 person-years (/100py) well prior to the index admission, more than tripled during the index period, then dropped to about 120/100py. For non-acute DDI participants, rates rose from 60/100py to under 110/100py by the index admission, then stabilised at about 90/100py thereafter. For non-DDI controls, dispensing rates grew slowly and consistently across the ten years from about 40/100py to almost 60/100py.

### Dispensing rates (DRs)

When just three periods 2007–2016 were modelled, *Model A* shows the DR for the pre-index period (mean 4.7 years) was 84/100py for acute DDI participants, 75/100py for non-acute DDI, and 47/100py for non-DDI participants (Table 2). For the index period, *Model B* shows the DR for acute DDI participants reached 325/100py, non-acute 108/100py, and 54/100py in non-DDI participants. After the index date (mean 3.7 years), *Model C* shows the DR in acute DDI participants stabilised at 141/100py, over twice that for non-DDI participants.

**Table 2:** Dispensing rates per 100 person-years, dispensing rate difference and dispensing rate ratio of antibiotics in the periods A) before B) about, and C) after index date.

	Dispensing rates (95%CI)*	Dispensing rate difference (95%CI)*	Dispensing rate ratio (95%CI)
**Model A: from July 2007 to 3 months before index date** (mean follow-up: 4.7 years)			
Acute DDI	84 (82 to 86)	37 (35 to 39)	1.79 (1.75 to 1.82)
Non-Acute DDI	75 (73 to 76)	28 (26 to 29)	1.59 (1.56 to 1.62)
Non-DDI	47 (47 to 48)	0	1.00 (ref.)
**Model B: for period from 3 months before to 3 months after index date** (mean follow-up: 0.5 years)			
Acute DDI	325 (317 to 334)	271 (263 to 280)	6.04 (5.90 to 6.19)
Non-Acute DDI	108 (106 to 111)	54 (52 to 57)	2.01 (1.97 to 2.06)
Non-DDI	54 (53 to 55)	0	1.00 (ref.)
**Model C: for period from 3 months after index date to June 2016** (mean follow-up: 3.7 years)			
Acute DDI	141 (138 to 145)	81 (79 to 84)	2.36 (2.31 to 2.41)
Non-Acute DDI	99 (97 to 101)	39 (37 to 41)	1.65 (1.63 to 1.68)
Non-DDI	60 (59 to 61)	0	1.00 (ref.)

Notes:

*Expressed per 100 person-years, and adjusted for year of birth, index year, gender, ethnic group and region

CI = confidence intervals, DDI = residents with diverticular disease of the intestine, non-DDI = matched controls without disease, Index date = date of first diverticular disease admission for DDI and their matched non-DDI participants.

### Dispensing rate differences (DRDs)

In each period, adjusted DRDs were markedly higher in DDI participants than in non-DDI participants (Table 2). Following the index period, the absolute excess in community dispensing rates for acute DDI participants was 81/100py, and for non-acute DDI participants 39/100py, higher than non-DDI counterparts, indicating additional dispensing of 55.8/100py overall (81*0.41 acute + 39*0.59 non-acute).

### Dispensing rate ratios (DRRs)

Table 2 also shows that acute DDI participants use antibiotics at 1.79 times the rate of non-DDI controls prior to their index event. For years post-index date, dispensing rates for acute DDI are 2.36 times higher, and for non-acute DDI 1.65 times higher; in combination this is more than double their non-DDI counterparts.

For these and the models, differences between DDI and non-DDI groups were significant with p-values <0.0001. Sensitivity analyses using conditional negative binomial methods yielded similar results.

## Discussion

### Summary

Routinely-collected data enabled estimates of community-dispensed antibiotic use in virtually a whole population. Following the index admission of DDI, 19% were hospitalised again for DDI, but they continued to used antibiotics well above the “norm” of under 60/100py experienced by their non-DDI peers, indicating a high level of primary care management of those with DDI.

This higher use of antibiotics is for all causes in both DDI groups. People with previous DDI may in general experience chronic conditions more frequently, be more likely to access help for symptoms, request antibiotics more readily, or their general practitioners may prescribe them antibiotics more often. If this were not so, then differences in dispensing could be a useful measure of DDI recurrence which typically measures re-hospitalisations alone.

Our results indicate that primary care-managed true or clinically-suspected recurrence is significant, and could be considered for burden of disease studies. Studies of recurrence that additionally include home-based follow-up also report higher recurrence than when re-admissions only are included.[30–32] For example, a multicentre randomised trial reported 38.5% recurrence inside 2 years (with strict diagnostic criteria that may underestimate “true” recurrence),[33] a cohort study reported 410 of 1045 (39.2%) experienced a recurrent event,[34] and a systematic review showed 43%-86% recurrence[30] although the time period may be unspecified.

Several other findings are notable. Firstly, dispensing rates were elevated years before the first hospital DDI diagnosis, suggesting that the index DDI diagnosis may follow prior episode(s) in which abdominal symptoms were suspected to be DDI but not recorded as such until subsequent hospital colonoscopy. Secondly, dispensing rates were extremely high near the index stay. This may represent dispensing for symptoms being treated shortly before the index hospitalisation; immediately post-discharge to complete a course of treatment; a new script to treat non-resolution of symptoms; or to provide “in reserve” supply of antibiotics for use when symptoms arise. None of these reasons, however, explains the ongoing elevated rates observed for years after the index stay.

### Strengths and limitations

This study accessed national databases of hospitalisations and dispensing over nine years, with robust p-values. Emergency department records were not accessed, so people presenting to emergency departments with DDI then discharged within three hours may have been misclassified as non-DDI, as may cases treated entirely within primary care, privately or prior to 2000. However with the healthcare system in NZ providing universal cover for its residents, and restrictions on access to antibiotics, admissions and dispensings are unlikely to be importantly impacted by selection bias. The indications for antibiotic dispensing are not collected in the national databases, so we cannot distinguish DDI dispensings from any others. While this could influence the internal validity of our findings, potential imbalance between groups is minimised by matching each DDI participant with 10 non-DDI participants. No adjustment for length of exposure was possible for people absent from NZ during the study period; models employed assume that residents remain in the cohort until death. Any biases introduced because of these limitations would alter only slightly the overall conclusions.

The findings depend upon reasonably accurate hospital coding of DDI diagnoses. We offer no NZ evidence of diagnosis coding quality. Elsewhere, the first three ICD-10 characters coded (K57) reliably record DDI diagnoses, but sub-codes less so,[35, 36] hence our decision not to differentiate e.g. between uncomplicated and complicated DDI in our reporting.

### Comparison with existing literature

Studies of DDI report recurring pain and other chronic symptoms, anxiety or fear of recurrence, all leading to lower quality of life.[1–3] Indeed, the proposal that DDI is a chronic condition[31] appears accepted with little debate, yet few studies inform management of patients presenting to primary care with presumed acute DDI.[12] We reviewed 14 clinical guidelines published since 2010 (11 using published evidence,[8, 37–46] three based on consensus[47–49]). While several recommend that patients who present to hospital with uncomplicated DDI can be managed as outpatients, just three mention primary care: one regarding initial presentations to primary care,[40] one regarding referrals to secondary care,[37] and one in the context of management of recurrent episodes.[46] While it is easy to infer that DDI is almost always managed in hospital, we have shown that recurrent DDI is commonly managed in primary care, with little supporting evidence. We therefore recommend that DDI guidelines should either include all relevant specialist groups—including primary care practitioners—or else clarify the setting(s) for which the guideline is intended.

### Implications for research and practice

Prior to the current study, we conducted semi-structured interviews with a small sample of volunteers with self-reported ongoing DDI. Their expressed needs were to reduce recurrence, better recognise symptoms, understand familial risk, intervene effectively and inform treatment for other conditions given their DDI (unpublished, details available from corresponding author). Similar needs are apparent also in the USA.[3] Evidence about risk factors, triggers, early signs of recurrence, effectiveness of potential alternatives to antibiotic therapy such as self-managed bowel rest, and when antibiotics or dietary modifications are advisable, would all be helpful.

Recognised good practice for people with recurring or chronic conditions is that they are involved with their own care plan.[50, 51] Improved self-management of chronic conditions is proposed as one strategy to slow rapidly rising global demand on health services as a result of population ageing.[52, 53] Modifying primary care management has potential to reduce hospital presentations, recurrence, antibiotic use (important with concerns about increasing antibiotic resistance)[18]. Easier access to diagnostic tools (including CT scan and quick point-of-care CRP testing) within primary care to exclude either complicated DDI or acute DDI as a diagnosis,[54] and developing and applying better prognostic rules[55] have potential in this regard. Results of recent studies in secondary care have led to guidelines increasingly avoiding antibiotics for uncomplicated DDI. Caution is needed before implementing similar advice in primary care, given our findings of widespread use of antibiotics and in the absence of readily available and reliable diagnostic testing (for example CT or CRP testing).[11]

Clinical guidance for those managing people with hospital-diagnosed DDI has previously overlooked their markedly higher rates of oral antibiotics use spanning at least several years that suggests DDI has greater impact on subsequent management than previously recognised. Evidence is needed to inform primary care physicians and their patients.

## References

[1] Simpson J, Neal KR, Scholefield JH, Spiller RC. Patterns of pain in diverticular disease and the influence of acute diverticulitis. Eur J Gastroenterol Hepatol. 2003;15(9):1005–10. Epub 2003/08/19. . 12923374 10.1097/00042737-200309000-00011

[2] Peery AF, Barrett PR, Park D, Rogers AJ, Galanko JA, Martin CF, et al A high-fiber diet does not protect against asymptomatic diverticulosis. Gastroenterol. 2012;(142):266–72. 10.1053/j.gastro.2011.10.035 22062360 PMC3724216

[3] Spiegel BM, Reid MW, Bolus R, Whitman CB, Talley J, Dea S, et al Development and validation of a disease-targeted quality of life instrument for chronic diverticular disease: the DV-QOL. Qual Life Res. 2015;24(1):163–79. 10.1007/s11136-014-0753-1 . 25059533

[4] Everhart JE, Ruhl CE. Burden of digestive diseases in the United States Part II: Lower gastrointestinal diseases. Gastroenterol. 2009;136(3):741–54. Epub 2009/01/27. 10.1053/j.gastro.2009.01.015 . 19166855

[5] Papagrigoriadis S, Debrah S, Koreli A, Husain A. Impact of diverticular disease on hospital costs and activity. Colorectal Dis. 2004;6(2):81–4. Epub 2004/03/11. 10.1111/j.1463-1318.2004.00532.x . 15008903

[6] Bollom A, Austrie J, Hirsch W, Nee J, Friedlander D, Ellingson K, et al Emergency department burden of diverticulitis in the USA, 2006–2013. Dig Dis Sci. 2017;62(10):2694–703. Epub 2017/03/24. 10.1007/s10620-017-4525-y . 28332105 PMC5610055

[7] Maconi G, Carmagnola S, Guzowski T. Intestinal ultrasonography in the diagnosis and management of colonic diverticular disease. J Clin Gastroenterol. 2016;50 Suppl 1:S20–2. Epub 2016/09/14. 10.1097/MCG.0000000000000657 . 27622354

[8] Stollman N, Smalley W, Hirano I, Committee AGAICG. American Gastroenterological Association Institute Guideline on the Management of Acute Diverticulitis. Gastroenterol. 2015;149(7):1944–9. Epub 2015/10/11. 10.1053/j.gastro.2015.10.003 . 26453777

[9] Viniol A, Keunecke C, Biroga T, Stadje R, Dornieden K, Bosner S, et al Studies of the symptom abdominal pain—a systematic review and meta-analysis. Fam Pract. 2014;31(5):517–29. Epub 2014/07/06. 10.1093/fampra/cmu036 . 24987023

[10] Okkes IM, Oskam SK, Lamberts H. The probability of specific diagnoses for patients presenting with common symptoms to Dutch family physicians. J Fam Pract. 2002;51(1):31–6. Epub 2002/04/03. . 11927060

[11] Wensaas KA, Hungin AP. Diverticular disease in the primary care setting. J Clin Gastroenterol. 2016;50:S86–S8. 10.1097/MCG.0000000000000596 27622376

[12] Whatling PJ. Diverticulosis and diverticular disease: Current concepts. InnovAiT. 2017;10(5):262–8.

[13] van de Wall BJM. Current Status of Treatment for Diverticulitis. Enschede, Netherlands: University of Twente; 2013.

[14] Ubaldi E, Grattagliano I, Lapi F, Pecchioli S, Cricelli C. Overview on the management of diverticular disease by Italian General Practitioners. Dig Liver Dis. 2019;51(1):63–7. Epub 2018/08/26. 10.1016/j.dld.2018.07.015 . 30143468

[15] Siddiqui J, Zahid A, Hong J, Young CJ. Colorectal surgeon consensus with diverticulitis clinical practice guidelines. World Journal of Gastrointestinal Surgery. 2017;9(11):224–32. Epub 2017/12/12. 10.4240/wjgs.v9.i11.224 . 29225733 PMC5714804

[16] Rosenlund IM, Leivseth L, Forde OH, Revhaug A. Regional variation in hospitalizations and outpatient appointments for diverticular disease in Norway: a nationwide cross-sectional study. Scand J Gastroenterol. 2018;53(10–11):1228–35. Epub 2018/09/29. 10.1080/00365521.2018.1506047 . 30265178

[17] De Bastiani R, Sanna G, Fracasso P, D’Urso M, Benedetto E, Tursi A. The management of patients with diverticulosis and diverticular disease in primary care: An online survey among Italian general pratictioners. J Clin Gastroenterol. 2016;50 Suppl 1:S89–92. Epub 2016/09/14. 10.1097/MCG.0000000000000580 . 27622377

[18] World Health Organization. Global Action Plan On Antimicrobial Resistance. Geneva World Health Organization; 2015. 10.7196/samj.9644 26242647

[19] Jaung R, Robertson J, Rowbotham D, Bissett I. Current management of acute diverticulitis: a survey of Australasian surgeons. N Z Med J. 2016;129(1431):23–9. Epub 2016/03/24. . 27005870

[20] Ministry of Health. National Health Index Wellington: Ministry of Health; 2017 [cited 2017 23 June]. www.health.govt.nz/our-work/health-identity/national-health-index.

[21] Ministry of Health. National Minimum Dataset (hospital events) Wellington: Ministry of Health; 2015 [cited 2017 20 July]. www.health.govt.nz/nz-health-statistics/national-collections-and-surveys/collections/national-minimum-dataset-hospital-events.

[22] Ministry of Health. ICD-10-AM/ACHI/ACS development Wellington: Ministry of Health; 2015 [cited 2017 5 December 2017].

[23] Ministry of Health. Pharmaceutical Collection Welington: Ministry of Health; 2012 [cited 2017 23 June]. www.health.govt.nz/nz-health-statistics/national-collections-and-surveys/collections/pharmaceutical-collection.

[24] Crowe FL, Balkwill A, Cairns BJ, Appleby PN, Green J, Reeves GK, et al Source of dietary fibre and diverticular disease incidence: a prospective study of UK women. Gut. 2014;63(9):1450–6. Epub 2014/01/05. 10.1136/gutjnl-2013-304644 . 24385599 PMC4145436

[25] Frieri G, Pimpo MT, Scarpignato C. Management of colonic diverticular disease. Digestion. 2006;73(SUPPL. 1):58–66. 16498253 10.1159/000089780

[26] Schechter S, Mulvey J, Eisenstat TE. Management of uncomplicated acute diverticulitis: results of a survey. Dis Colon Rectum. 1999;42(4):470–5; discussion 5–6. Epub 1999/04/24. . 10215046 10.1007/BF02234169

[27] Murphy T, Hunt RH, Fried M, Krabshuis JH. World Gastroenterology Organisation Practice Guidelines: Diverticular Disease. World Gastroenterology Organisation 2007.

[28] Jacobs DO. Diverticulitis. The New England Journal of Medicine. 2007;357:2057–66. 10.1056/NEJMcp073228 18003962

[29] Pearce N. Analysis of matched case-control studies. Br Med J. 2016;352:i969 Epub 2016/02/27. 10.1136/bmj.i969 . 26916049 PMC4770817

[30] Peppas G, Bliziotis IA, Oikonomaki D, Falagas ME. Outcomes after medical and surgical treatment of diverticulitis: a systematic review of the available evidence. J Gastroenterol Hepatol. 2007;22(9):1360–8. Epub 2007/08/25. 10.1111/j.1440-1746.2007.05118.x . 17716342

[31] Strate LL, Modi R, Cohen E, Spiegel BMR. Diverticular disease as a chronic illness: evolving epidemiologic and clinical insights. Am J Gastroenterol. 2012;107(10):1486–93. 10.1038/ajg.2012.194 22777341

[32] Shah SD, Cifu AS. Management of acute diverticulitis. J Am Med Assoc. 2017;318(3):291–2. 10.1001/jama.2017.6373 28719679

[33] Kruis W, Kardalinos V, Eisenbach T, Lukas M, Vich T, Bunganic I, et al Randomised clinical trial: mesalazine versus placebo in the prevention of diverticulitis recurrence. Aliment Pharmacol Ther. 2017;46(3):282–91. 10.1111/apt.14152 . 28543263 PMC5518301

[34] Bharucha AE, Parthasarathy G, Ditah I, Fletcher JG, Ewelukwa O, Pendlimari R, et al Temporal trends in the incidence and natural history of diverticulitis: A population-based study. Am J Gastroenterol. 2015;110(11):1589–96. 10.1038/ajg.2015.302 . 26416187 PMC4676761

[35] Erichsen R, Strate L, Sorensen HT, Baron JA. Positive predictive values of the International Classification of Disease, 10th edition diagnoses codes for diverticular disease in the Danish National Registry of Patients. Clin Exp Gastroenterol. 2010;3:139–42. 10.2147/CEG.S13293 . 21694857 PMC3108666

[36] Tan A. 0323: The accuracy of clinical coding for diverticular disease and the implication for trainees intending to undertake research. Int J Surg. 2013;11(8):689.

[37] Fozard JB, Armitage NC, Schofield JB, Jones OM, Association of Coloproctology of Great Britain and Ireland. ACPGBI position statement on elective resection for diverticulitis. Colorectal Dis. 2011;13 Suppl 3:1–11. Epub 2011/03/05. 10.1111/j.1463-1318.2010.02531.x . 21366820

[38] Maconi G, Barbara G, Bosetti C, Cuomo R, Annibale B. Treatment of diverticular disease of the colon and prevention of acute diverticulitis: a systematic review. Dis Colon Rectum. 2011;54(10):1326–38. Epub 2011/09/10. 10.1097/DCR.0b013e318223cb2b . 21904150

[39] Andersen JC, Bundgaard L, Elbrond H, Laurberg S, Walker LR, Stovring J, et al Danish national guidelines for treatment of diverticular disease. Danish Med J. 2012;59(5):C4453 . 22549495

[40] Andeweg CS, Mulder IM, Felt-Bersma RJ, Verbon A, van der Wilt GJ, van Goor H, et al Guidelines of diagnostics and treatment of acute left-sided colonic diverticulitis. Dig Surg. 2013;30(4–6):278–92. Epub 2013/08/24. 10.1159/000354035 . 23969324

[41] Feingold D, Steele SR, Lee S, Kaiser A, Boushey R, Buie WD, et al Practice parameters for the treatment of sigmoid diverticulitis. Dis Colon Rectum. 2014;57(3):284–94. Epub 2014/02/11. 10.1097/DCR.0000000000000075 . 24509449

[42] Kruis W, Germer CT, Leifeld L. Diverticular disease: guidelines of the German Society for Gastroenterology, digestive and metabolic diseases and the German Society for General and Visceral Surgery. Digestion. 2014;90(3):190–207. Epub 2014/11/22. 10.1159/000367625 . 25413249

[43] Morris AM, Regenbogen SE, Hardiman KM, Hendren S. Sigmoid diverticulitis: a systematic review. J Am Med Assoc. 2014;311(3):287–97. Epub 2014/01/17. 10.1001/jama.2013.282025 . 24430321

[44] Pietrzak A, Bartnik W, Szczepkowski M, Krokowicz P, Dziki A, Regula J, et al Polish interdisciplinary consensus on diagnostics and treatment of colonic diverticulosis (2015). Pol Przegl Chir. 2015;87(4):203–20. Epub 2015/07/07. 10.1515/pjs-2015-0045 . 26146121

[45] Schreyer AG, Layer G. S2k guidlines for diverticular disease and diverticulitis: diagnosis, classification, and therapy for the radiologist. RöFo-Fortschritte auf dem Gebiet der Röntgenstrahlen und der bildgebenden Verfahren: Georg Thieme Verlag KG; 2015. p. 676–84. 10.1055/s-0034-1399526 26019048

[46] Boermeester MA, Humes DJ, Velmahos GC, Søreide K. Contemporary review of risk-stratified management in acute uncomplicated and complicated diverticulitis. World J Surg. 2016;40(10):2537–45. Epub 2016/05/22. 10.1007/s00268-016-3560-8 . 27206400

[47] Binda GA, Cuomo R, Laghi A, Nascimbeni R, Serventi A, Bellini D, et al Practice parameters for the treatment of colonic diverticular disease: Italian Society of Colon and Rectal Surgery (SICCR) guidelines. Tech Coloproctol. 2015;19(10):615–26. Epub 2015/09/18. 10.1007/s10151-015-1370-x . 26377584

[48] O’Leary DP, Lynch N, Clancy C, Winter DC, Myers E. International, expert-based, consensus statement regarding the management of acute diverticulitis. JAMA Surg. 2015;150(9):899–904. Epub 2015/07/16. 10.1001/jamasurg.2015.1675 . 26176318

[49] Sartelli M, Catena F, Ansaloni L, Coccolini F, Griffiths EA, Abu-Zidan FM, et al WSES Guidelines for the management of acute left sided colonic diverticulitis in the emergency setting. World J Emerg Surg. 2016;11:37 Epub 2016/08/02. 10.1186/s13017-016-0095-0 . 27478494 PMC4966807

[50] Coulter A, Entwistle VA, Eccles A, Ryan S, Shepperd S, Perera R. Personalised care planning for adults with chronic or long-term health conditions. Cochrane Database Syst Rev. 2015;3:CD010523 Epub 2015/03/04. 10.1002/14651858.CD010523.pub2 . 25733495 PMC6486144

[51] Reed RL, Roeger L, Howard S, Oliver-Baxter JM, Battersby MW, Bond M, et al A self-management support program for older Australians with multiple chronic conditions: a randomised controlled trial. Med J Aust. 2018;208(2):69–74. Epub 2018/02/02. . 29385967 10.5694/mja17.00127

[52] Brady TJ, Anderson LA, Kobau R. Chronic disease self-management support: public health perspectives. Front Public Health. 2014;2:234 Epub 2014/01/01. 10.3389/fpubh.2014.00234 . 25964925 PMC4410343

[53] Honey ML, Roy DE, Bycroft JJ, Boyd MA. New Zealand consumers’ health information needs: results of an interpretive descriptive study. J Prim Health Care. 2014;6(3):203–11. Epub 2014/09/10. . 25194247

[54] Tan JP, Barazanchi AW, Singh PP, Hill AG, Maccormick AD. Predictors of acute diverticulitis severity: A systematic review. Int J Surg. 2016;26:43–52. Epub 2016/01/19. 10.1016/j.ijsu.2016.01.005 . 26777741

[55] Jamal Talabani A, Endreseth BH, Lydersen S, Edna TH. Clinical diagnostic accuracy of acute colonic diverticulitis in patients admitted with acute abdominal pain, a receiver operating characteristic curve analysis. Int J Colorectal Dis. 2017;32(1):41–7. Epub 2016/09/11. 10.1007/s00384-016-2644-0 . 27613727 PMC5219887

